# Supplementary material for: Discovery of entanglement generation by elastic collision to realise the original Einstein-Podolsky-Rosen thought experiment
Source: npj Quantum Inf. 2025 May 14;11(1):76. doi: 10.1038/s41534-025-01028-7 (PMC12078181; doi:10.1038/s41534-025-01028-7)
Supplement: Supplementary file 1 — Supplementary Information [file 41534_2025_1028_MOESM1_ESM.pdf]

Discovery of entanglement generation by elastic collision to realise the original Einstein-Podolsky-Rosen thought experiment

— Supplementary Information —

Roman Schnabel
[1] *Institut für Quantenphysik & Zentrum für Optische Quantentechnologien, Universität Hamburg, Luruper Chaussee 149, 22761 Hamburg, Germany**
(Dated: May 1st, 2025, corrected illustrations on June 19th, 2025)

ILLUSTRATING THE ENTANGLING COLLISION IN PHASE SPACE

The physical content of the figure of the main manuscript can alternatively be represented in the phase space of positions and momenta, see Fig. 1 below. Such a phase space representation corresponds to the usual quantum mechanically complete representation.

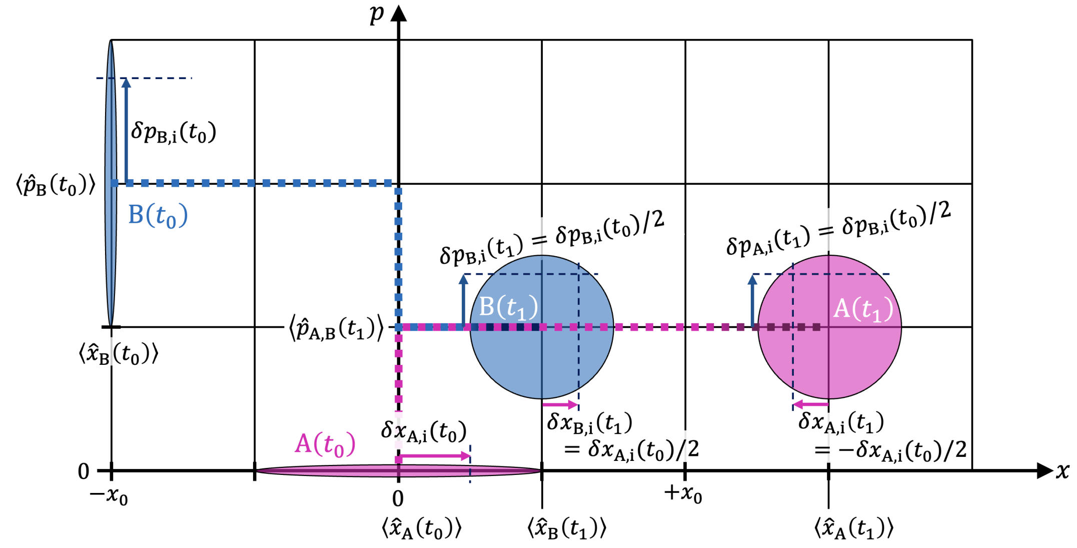

FIG. 1: **EPR entanglement from conventional elastic collision in phase space** – Shown are expectation values surrounded by Gaussian quantum uncertainties (thin uncertainty areas) of the positions and momenta of two bodies A and B at time t_0 shortly before their collision at position $\langle x_{\mathrm{A}}(t_0)\rangle$, as well as at time t_1 shortly after their collision (large uncertainty areas). The two thin ellipses represent squeezed, mutually uncorrelated minimal uncertainties before the collision. The two large uncertainty areas represent EPR entangled uncertainties after the collision. Individually, they are not minimal. The entanglement-generating collision happens at time $\langle \hat{t}_{\mathrm{coll}}\rangle$ with $t_0 < \langle \hat{t}_{\mathrm{coll}}\rangle < t_1$. (The calculation in the main text shows that the time of the collision must actually be described by an "arrival time" operator [1, 2].) The bodies' mass ratio is $m_{\mathrm{B}} = 3m_{\mathrm{A}}$, due to which the initial momentum of B is equally distributed amongst A and B. The distribution of the momentum of B is independent of its magnitude, from which it follows that the momentum *uncertainty* of B is also distributed equally. With the approximation that the initial momentum uncertainty of A is negligible, the momentum uncertainties after the collision are perfectly quantum correlated. Subscript 'i' represents a single measurement example. The positions after the collision are determined as follows: B's velocity is halved, while A's velocity is three-times larger than that of B. This determines the position expectation values at time t_1 as shown. The large position uncertainty of A, however, creates a collision timing uncertainty. If the collision already takes place when B reaches the left end of A's ellipse of uncertainty, body A gains speed earlier and is at the right end of the circular uncertainty at time t_1. In contrast, the body B has its reduced velocity earlier and ends up at the left end of its uncertainty circle. The spatial uncertainty after the collision are perfectly quantum-anticorrelated, if one again makes the approximation that the initial squeezed spatial uncertainty of B is negligible. *The figure makes obvious why EPR correlations are observed. By measuring either A or B we can predict with certainty, and without in any way disturbing the second system, either the value of x or the value of p.*

MASS RATIO 1:3

Case (i): The lighter mass ('A') initially has zero momentum and a strongly squeezed momentum uncertainty. Here, every collision 'i' of an ensemble of identical experiments with 50% momentum transfer is described by

$$\frac{1}{2} m_{\rm B} v_{\rm B,i}(t_0) = m_{\rm B} v_{\rm B,i}(t_1) = m_{\rm A} v_{\rm A,i}(t_1)\,.$$

Energy conservation enforces

$$m_{\rm B} v^2_{\rm B,i}(t_0) = m_{\rm B} v^2_{\rm B,i}(t_1) + m_{\rm A} v^2_{\rm A,i}(t_1)\,,$$

from which follows

$$m_{\rm B} = 3\, m_{\rm A}\,.$$

Case (ii): The heavier mass 'A' initially has zero momentum and a strongly squeezed momentum uncertainty. Here, every collision 'i' of an ensemble of identical experiments with 50% momentum transfer involves a momentum sign flip and is described by

$$\begin{aligned} \frac{1}{2} p_{\rm B,i}(t_0) &= -p_{\rm B,i}(t_1)\,, \\ p_{\rm B,i}(t_0) &= p_{\rm B,i}(t_1) + p_{\rm A,i}(t_1)\,, \\ \Rightarrow \quad \frac{1}{2} m_{\rm B} v_{\rm B,i}(t_0) &= -m_{\rm B} v_{\rm B,i}(t_1) = \frac{1}{3} m_{\rm A} v_{\rm A,i}(t_1)\,. \end{aligned}$$

Energy conservation enforces in this case

$$m_{\rm B} = \frac{1}{3} m_{\rm A}\,.$$

A one dimensional elastic collision between two particles with mass ratio 1:3 results in a 50% momentum transfer. It corresponds to a balanced redistribution of momentum expectation values as well as momentum uncertainties. The redistribution happens in both directions, from 'A' to 'B' and vice versa. However, if one particle has negligible (strongly squeezed) momentum uncertainty, the momentum transfer is de facto one way. Such a collision produces maximal quantum correlation of the particles' momenta if the mass ratio is 1:3.

* Electronic address: roman.schnabel@uni-hamburg.de

[1] J. Kijowski, "On the time operator in quantum mechanics and the Heisenberg uncertainty relation for energy and time", *Reports on Mathematical Physics*, vol. 6, 361–386, 1974.

[2] J. Kiukas, A. Ruschhaupt, P. O. Schmidt, and R. F. Werner, "Exact energy–time uncertainty relation for arrival time by absorption", *J. Phys. A: Math. Theor.*, vol. 45, 185301, 2012.
